# Hypomethylating Agents (HMAs) as Salvage Therapy in Relapsed or Refractory AML: An Italian Multicentric Retrospective Study

**DOI:** 10.3390/biomedicines9080972

**Published:** 2021-08-06

**Authors:** Federica Lessi, Marica Laurino, Cristina Papayannidis, Orsola Vitagliano, Francesco Grimaldi, Davide Lazzarotto, Michele Gottardi, Elena Crisà, Marta Riva, Gianluigi Reda, Mario Ermani, Gianpietro Semenzato, Livio Trentin, Felicetto Ferrara

**Affiliations:** 1 Hematology Unit, Department of Medicine (DIMED), Azienda Ospedale Università Padova, 35100 Padova, Italy; maricalaurino@gmail.com (M.L.); g.semenzato@unipd.it (G.S.); 2 Department of Hematology and Oncology “L. and A. Seràgnoli” S.Orsola Malpighi University Hospital, 40100 Bologna, Italy; cristina.papayannidis@unibo.it; 3 Division of Hematology, Cardarelli Hospital, 80100 Naples, Italy; orsola.vitagliano@gmail.com (O.V.); felicetto.ferrara@aocardarelli.it (F.F.); 4 Department of Medicina Clinica e Chirurgia, AOU Federico II di Napoli, 80100 Naples, Italy; grimaldi.francesco@gmail.com; 5 Hematology and SCT Unit, University of Udine, Azienda Sanitaria Universitaria Integrata di Udine, 33100 Udine, Italy; davide.lazzarotto@asuiud.sanita.fvg.it; 6 Hematology, Treviso Hospital, 31100 Treviso, Italy; michele.gottardi@iov.veneto.it; 7 Division of Hematology, Department of Translational Medicine, Università del Piemonte Orientale and Ospedale Maggiore della Carità, 28100 Novara, Italy; elena.crisa@med.uniupo.it; 8 Hematology, ASST Grande Ospedale Metropolitano Niguarda, 20162 Milan, Italy; marta.riva@ospedaleniguarda.it; 9 Hematology, Fondazione IRCCS Ca’ Granda Ospedale Maggiore Policlinico di Milano, 20162 Milano, Italy; gianluigi.reda@policlinico.mi.it; 10 Statistic and Informatics Unit, Department of Neurosciences, School of Medicine, University of Padua, 35100 Padua, Italy; mario.ermani@unipd.it

**Keywords:** azacytidine, decitabine, HMA, relapsed, refractory, acute myeloid leukemia

## Abstract

Data on the use of azacytidine and decitabine as salvage therapy for acute myeloid leukemia are limited. We retrospectively reviewed clinical records of 100 patients treated with hypomethylating agents (HMA) as salvage therapy in nine Italian institutions. A total of 24% of patients obtained a response to HMA (CR, PR, or CRi), while 26% showed a stable disease (SD); 50% of patients experienced progressive disease. Median OS was 6.5 months. OS in patients with de novo AML was 6.1 months, while OS in patients with secondary AML (sAML) was 12.3 months (*p* = 0.037). Median OS after HMA in patients with SD as best response to HMA was similar to median OS in patients with response to HMA (10.6 months vs. 13 months). On multivariate analysis, OS difference between patients who obtained a response versus patients who did not was significant (*p* = 0.0037). OS difference in sAML was significantly better than in de novo AML (*p* < 0.00001). HMA showed a remarkable efficacy in terms of response rate and OS in a subgroup of patients (sAMLs), historically characterized by a poor outcome. Therefore, 5Azacitidine and decitabine may represent a good clinical option in a selected patient population with relapsed or refractory AML, unsuitable for allo-HSCT.

## 1. Introduction

Relapsed AML is a potentially curable disease, but results of salvage therapy are generally dismal, with median survival of 4–6 months only, with a trend towards worse outcomes in patients aged ≥60 year [1], and no standard of care defined, especially when allo-HSCT is not a suitable option. This may be explained by concerns regarding excessive toxicity of available treatments, AML resistance related to more frequent adverse karyotype and/or secondary AML, and involvement of chemo-refractory early hemopoietic precursors in the pathogenesis of the disease. Overall, the cumulative incidence of relapse is about 60% at three years for patients in the European LeukemiaNet (ELN) favorable-risk category and exceeds 85% for those in the adverse-risk category [2,3,4]. Hypomethylating agents (HMAs) are nowadays widely used for frontline therapy in elderly patients, unfit for conventional chemotherapy, with good results in terms of response rate, OS and quality of life [5,6,7,8,9]. Nevertheless, the efficacy of these drugs in relapsed patients is not well defined, and mostly based on retrospective studies on limited patient cohorts [10,11,12], with only one large study involving about 1000 patients, showing a CR rate of 11%, and a median OS of 6.7 months [13]. Therefore, a data collection from nine Italian centers involved in AML patient treatment was performed, aimed at assessing efficacy of HMAs in a real life setting and really poor patient population.

## 2. Materials and Methods

Adult patients, affected by AML diagnosed according to 2016 World Health Organization (WHO) criteria, and previously treated with HMAs at relapse were retrospectively enrolled in the study. Disease status included relapsed AML (even after allogenic stem cell transplantation (alloHSCT)), and refractory leukemia to at least one course of intensive chemotherapy (IC). Patients who received HMA before alloHSCT and patients who received HMA as first-line therapy were excluded. Clinical and biological data referring to the period from 2006 to 2017 were collected by every center, and datasets were combined and analyzed by the coordinating center (Padua University Hospital, Padua, Italy). The study was approved by the institutional review board of Padua University Hospital (ID 4227/AO/17 on 6 July 2017) and was conducted in accordance with the Declaration of Helsinki. Relapsed AML was defined by the recurrence of >5% blasts in the peripheral blood and/or bone marrow of patients after achieving a complete remission (CR), treated with one or more lines of therapy [2]. Duration of the first CR was defined as the duration between CR achievement and the date of relapse. It was set to 0 in refractory AML patients. Clinical and laboratory data were collected at the time of diagnosis and at the beginning of HMA administration. Cytogenetics were classified according to 2017 ELN Recommendations [2]. Molecular biology data were collected when available. Additional data included: type of HMA and the administration regimen, including number of cycles, any concurrent therapy, performance status (ECOG) at the beginning of HMA, transfusion needs and hospitalization. The primary end point of the study was OS, whereas the secondary endpoints included rates of response, which were evaluated according to 2017 ELN Recommendations and were assigned by the investigator providing the data. Response duration was measured from the date of response to progression or death. OS was measured from time of initiation of HMAs until death or last follow-up.

Survival analysis was performed using Kaplan–Meier curves. Significance in survival time between groups was tested using log-rank test. In multivariate analysis, the Cox proportional hazard model was used to find the true independent factors. Significance was set at *p* < 0.05.

## 3. Results

One hundred AML patients were included in the study: 68 males and 32 females. Median age at diagnosis was 64.3 years. A total of 29% of patients were affected by secondary AML. One patient was AML with t(9;11), two were AML with t(8;21), one was AML with inv(3), one patient was AML with inv(16), twenty-seven were AML MRC, two were therapy-related AML, and sixty-one were AML NOS. According to ELN 2017 risk stratification, 10 patients were favorable risk, 50 were intermediate risk, and 33 were adverse risk. Cytogenetic risk was not available for seven patients. A total of 80 patients were treated with azacytidine (75 mg/sqm for 7 days every 28) and 20 with decitabine, at the standard dosage of 20 mg/sqm for 5 days every 28. A total of 60% of patients received HMA as second-line therapy for their disease, 29% as third-line and 11% were beyond third-line therapy. Among the 60 patients who received HMAs as second-line therapy 35% were 70 years old or older, 40% had a PS ECOG score of 2 or more, 36.7% had adverse cytogenetic risk; 40% of patients had at least one of the above-mentioned characteristics, 33.3% had two, 3.3% had three and only 23.3% had none. A total of 20% of patients underwent allogeneic stem cell transplantation before HMA, and 31.6% of these received concomitant donor lymphocyte infusions. All patients underwent intensive chemotherapy (i.e., FLAI or 3 + 7 like) as first-line induction. Performance status ECOG at the beginning of HMA was 0 for 19% of patients, 1 for 30% of patients, 2 for 41% of patients, and >2 for 10% of patients. A total of 54 patients had relapsed AML, and 44 patients had refractory AML. Data were not available for two patients. Median number of HMA courses was three (range 1–39). Patient characteristics are reported in Table 1.

**Figure 1:**
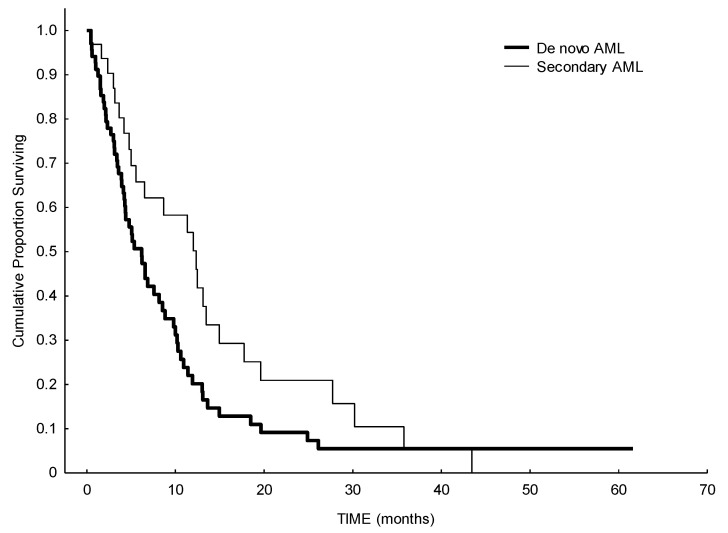
Survival analysis from starting of HMA in de novo versus secondary AML. Median survival in de novo AML was 6.1 months, median survival in secondary AML was 12.3 months (*p* < 0.0035).

**Figure 2:**
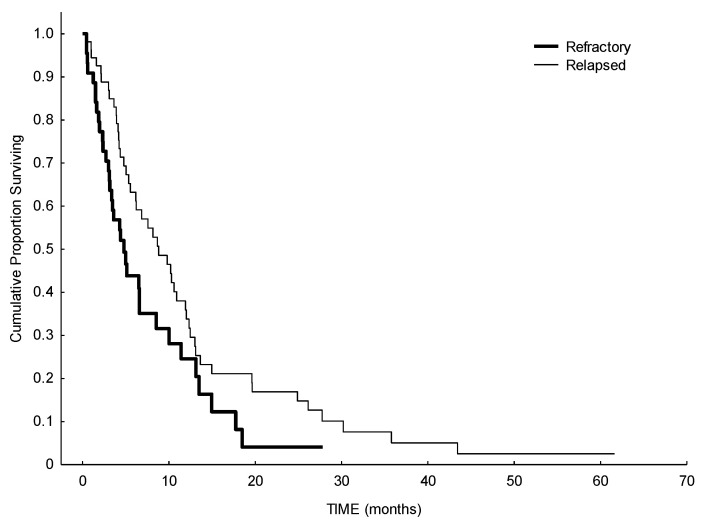
Survival analysis from starting of HMA in refractory versus relapsed AML. Median OS in patients with refractory disease was 4.8 months, while it was higher in patients with relapsed disease (8.8 months) (*p* = 0.055).

**Figure 3:**
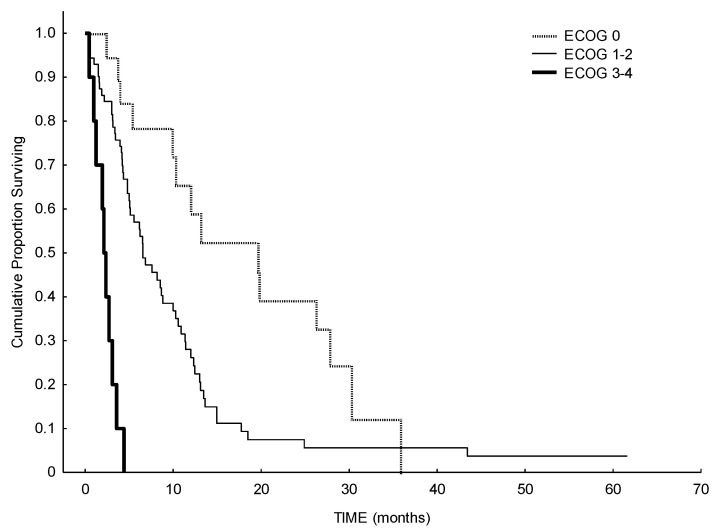
Survival analysis from starting of HMA according to ECOG performance status at relapse. Patients with ECOG 0 showed a median OS of 19.6 months, patients with ECOG 1–2 or 3–4, showed a median OS of 6.5 months and 2.1 months, respectively (*p* < 0.0001).

In terms of response, the ORR was 24% (including complete remission (CR), partial remission (PR) and CR with incomplete hematologic recovery (CRi)), 26% of the patients had a stable disease (SD), and 50% of patients showed no response to HMA. Median number of therapy courses to reach a response was four (range 1–9). A total of 50% of patients with adverse cytogenetic risk and 42% of patients with favorable/intermediate cytogenetic risk reached a response or a stable disease with HMA. Median OS of the whole cohort was 6.5 months, with a statistically significant difference between de novo AML (6.1 months) and secondary AML (12.3 months) (*p* = 0.037). (Figure 1). Considering the impact of previous treatments received before HMA, median OS in patients with refractory disease was 4.8 months, while it was higher in patients with relapsed disease (8.8 months) (*p* = 0.055) (Figure 2). We observed an effect of delay of relapse: patients relapsing more than 10 months after first-line therapy have a median survival of 9.8 months, while patients relapsing before 10 months have a median survival of 5.3 months after HMA (log-rank, *p* = 0.03). As expected, a better performance status may influence a better outcome: patients with ECOG 0 at the beginning of HMA showed a median OS of 19.6 months, while patients with ECOG 1–2 or 3–4, showed a median OS of 6.5 months and 2.1 months, respectively (*p* < 0.00001) (Figure 3).

Median OS after HMA in patients with SD as best response to HMA was similar to median OS in patients who obtained a response to HMA (10.6 months vs. 13 months), while OS in unresponsive patients was 3.3 months (*p* < 0.00001). On multivariate analysis, OS differences between: 1) secondary AML and de novo AML; 2) SD and responsive group vs. progressive disease group; and 3) patients with ECOG 0 and >1 remained significant. Hazard ratio for death for secondary AML vs. de novo AML was 1.84 (CI 95%, *p* = 0.018); for ECOG 1–2 patients vs. ECOG 0 patients it was 3.1; and for ECOG 0 vs. ECOG 3–4 it was 6.2, (*p* < 0.001, CI 95%).

Age at relapse, previous ASCT, bone marrow blast rate, peripheral WBC at relapse, ELN 2017 risk score, antibiotic or antifungal prophylaxis, and WHO classification were not significant variables for OS.

Median number of hospitalization days during HMA was 7.5 days (range 1–55), with 56% of patients never having been hospitalized. A total of 88% of patients did not experience any major infections during treatment (defined as grade 3 or worse according to CTCAE), 8% of patients experienced one major infective event and only 3% experienced three or more major infective events. Regarding fungal infections, 91% of patients did not experience any of them, 7% had possible and 1% proven infection. We did not collect any probable fungal infections in our data set. A total of 58% of patients did not experience any episode of neutropenic fever, 21% experienced one, 12% experienced two and only 8% experienced three or more. We did not find any significant differences between packed red blood cells transfusion needs before and after best response to therapy, and we found a worsening in platelet transfusion needs after best response to HMA (mean 1.5 platelet concentrates before HMA, mean 2.4 after HMA, *p* = 0.0013).

## 4. Discussion

In this study we assessed the effect of HMAs in a real life setting and demonstrated that HMAs could be a therapeutic option in this poor risk population.

We observed that 24% of the overall population reached a response, which, as expected, is slightly lower than patients treated with decitabine (CR + CRi + PR + CRp = 30%) [7] and with azacytidine (CR + CRi + PR = 31.1%) [9] in the first-line setting. This slight effect might be mainly due to the heavily pretreated patient population involved in our study, in which mechanisms of clonal evolution and chemoresistance may influence a lower response rate. Nevertheless, as demonstrated in other studies which evaluate HMAs in AML [5,6,13,14], CR correlated favorably with OS, but the improvement of OS was observed even in PR and SD (in the larger retrospective study by Stahl et al. OS for CRs was 25.3 months, for CRis 14.6 months, and for SDs 10.4 months). As a consequence, the aim of treatment in elderly AML patients should not be primary focused, as for the young setting, on the strict achievement of a CR and the disease eradication, but should also include the maintenance of a good quality of life, which can be pursued and maintained also in patients with a persistent, but stable, leukemic burden disease [15]. OS of the whole cohort herein reported is consistent with data reported by other studies on the same patient population [1,3] and treated with HMA. To extend these observations we tried to investigate HMA efficacy in different subsets of the relapsed patients, in order to identify potential variables related to a better outcome. As previously reported by Ivanoff et al. [16] in a small study involving 47 patients, the authors identified late relapse (more than 12 months) after first-line chemotherapy as a factor possibly impacting on a better OS. This was confirmed in univariate analysis by Stahl et al. [13] but not in our study. We did not observe significantly better OS in patients with low WBC at relapse or with low bone marrow or peripheral blood blast rate, as in other studies [10,13,16], and we did not observe an impact on OS of cytogenetic risk groups. A good ECOG performance status (0–1) at relapse was significant for better OS, and it probably reflects lower toxic effects of previous chemotherapies. In our series, HMA response rate was better in secondary than in de novo AML, highlighting a subset population who may not benefit from intensive chemotherapy in the relapsed setting and for whom the question is still controversial in the first-line approach [14,17,18,19].

Mechanism of action of HMAs includes not only DNA methylation but also immunomodulating effects, as demonstrated by studies mainly in the setting of alloHSCT, involving an upregulated expression of tumor-associated antigens by leukemic blasts [20,21]. In our study this last effect may have influenced the positive results in our small cohort of previously transplanted patients. Even if we focused on relapsed not transplant eligible elderly patients, we may suggest a potential beneficial role of HMAs as a bridge to transplant option, alone or in combination with other novel agents (i.e., *IDH* inhibitors, *FLT3* inhibitors, venetoclax [22,23]), after relapse or refractoriness to previous chemotherapy, even in a younger setting.

As far as toxicity is concerned, most of the patients did not experience any infective event during treatment, probably due to wide use of antifungal and antibacterial prophylaxis in this population. This may explain the small rate of hospitalization, the good quality of life reported by patients and the consequent cost reduction. Further prospective studies on the quality of life assessment are required to confirm these observations.

## 5. Conclusions

HMAs could represent a good therapeutic option in a selected population of relapsed elderly AML patients, not suitable for alloHSCT, apparently regardless of cytogenetic risk, and for whom the preservation of a good quality of life should be privileged. Further studies are needed in order to define which is the right population who can benefit from these agents, what is their role in transplant eligible patients and whether these results can be affected by the combination of novel targeted agents.

## Figures and Tables

**Table 1:** Characteristics of study population (100 patients, median age 64.3 years (range 25.2–82.8)).

Characteristic		N (%)
Sex	M F	68 (68%) 32 (32%)
WHO 2016	AML with recurrent genetic abnormalities AML/MRC Therapy Related AML AML, NOS not available	5 (5%) 27 (27%) 2 (2%) 61 (61%) 5 (5%)
ELN2017 Risk score	Favorable Intermediate Adverse not available	10 (10%) 50 (0%) 33 (33%) 7 (7%)
Genetic mutations	*FLT3* *NPM1* *CEBPA*	7 (7%) 10 (10%) 2 2(2%)
Type of AML	Secondary AML De novo AML Relapsed AML Refractory AML not available	32 (32%) 68 (68%)
		54 (54%) 44 (44%) 2 (2%)
N° of previous lines of therapy before HMA	1 2 ≥3	60 (60%) 29 (29%) 11 (11%)
HMA	Azacytidine Decitabine	80 (80%) 20 (20%)
AlloSCT before HMA	YES NO	20 (20%) 80 (80%)
Response to HMA	Response (CR, PR or CRi) Stable disease (SD) No response	24 (24%) 26 (26%) 50 (50%)
